# A Nationwide Survey of Attitudes and Expectations Toward APOE4 Gene Testing in Adults: The APOE for You Survey

**DOI:** 10.1192/bjo.2026.11177

**Published:** 2026-06-30

**Authors:** Jerry Tan, Linda Pointon, Benjamin R Underwood, Ivan Koychev, Paresh Malhotra

**Affiliations:** 1 Department of Psychiatry, University of Oxford, Oxford, United Kingdom; 2 Oxford Health NHS Foundation Trust, Oxford, United Kingdom; 3 Department of Psychiatry, University ofCambridge, Cambridge, United Kingdom; 4 Cambridge and Peterborough NHS Foundation Trust, Cambridge, United Kingdom; 5 Department of Brain Health Sciences, Imperial College, London, United Kingdom

## Abstract

**Aims:**

The APOE-ε4 allele is the most significant genetic risk factor for developing late-onset Alzheimer’s disease (AD), with an estimated lifetime risk of 30% in ε4 heterozygotes. Previous guidelines have recommended against APOE genotyping based on its limited clinical utility in routine practice. This has changed with the advent of amyloid-targeting immunotherapies, multimodal interventions for reducing AD risk and commercially available APOE genotyping services. Given this changing landscape, there is a need to formulate APOE-ε4 genetic counselling tailored to the UK context. We set out to study the attitudes of UK adults to genetic testing and counselling for the APOE-ε4 gene.

**Methods:**

We carried out a digital survey on the online POrtal for Patient and Public Engagement in Research (POPPED, https://popped.org.uk). This was advertised via research register mailing lists and is still ongoing. The survey queried respondent demographics, motivations and concerns regarding APOE-ε4 testing, content and format of genetic counselling, and willingness to undertake preventative interventions. Responses were collected with a combination of 5-point Likert scales, ranking and multiple choice questions. Ethical approval was obtained from a university ethics committee and all respondents consented to have their responses stored anonymously on a secure server.

**Results:**

794 responses were received from 7 January – 4 February 2026. Most respondents were aged 65–74 years (38.7%) or 75–84 years (23%), 66.9% were female, and 61.8% had a family member with dementia. 82.7% of respondents were ‘likely’ or ‘some what likely’ to get tested for the APOE-ε4 gene. The main motivations were to contribute to research (85.4%), to adjust life plans accordingly (71.9%) or to understand their dementia risk (70.9%). The main concerns were that no cure for dementia exists (50.4%), inaccurate risk estimation (50.4%), and emotional upset (44.1%). The preferred format of genetic counselling was a face-to-face appointment (86.1% rated this as ‘very appropriate’), and 73.4% of respondents would be likely to accept subsequent preventative lifestyle interventions.

**Conclusion:**

A high proportion of our respondents would be likely to seek out APOE-ε4 testing and counselling, and to subsequently accept lifestyle interventions and be involved in research. While our sample may not be completely representative of the general population, these initial results suggest that there is a need to develop guidelines for face-to-face genetic counselling for UK adults who choose to undergo APOE-ε4 testing.

